# Urinary profiles of methoxyphenamine and its metabolite after inhalation of methoxyphenamine smoke in humans: aiming to distinguish between active and passive exposure

**DOI:** 10.1007/s11419-022-00658-2

**Published:** 2023-01-06

**Authors:** Haruka Morinaka, Asuka Kaizaki-Mitsumoto, Hokuto Morohoshi, Naoki Uchida, Satoshi Numazawa

**Affiliations:** 1 Division of Toxicology, Department of Pharmacology, Toxicology and Therapeutics, Showa University School of Pharmacy, 1-5-8 Hatanodai, Shinagawa-ku, Tokyo, 142-8555 Japan; 2 Clinical Research Institute for Clinical Pharmacology and Therapeutics, Showa University, 6-11-11 Kitakarasuyama, Setagaya-ku, Tokyo, 157-8577 Japan; 3 Department of Hygiene, Public Health and Preventive Medicine, Showa University School of Medicine, 1-5-8 Hatanodai, Shinagawa-ku, Tokyo, 142-8555 Japan; 4 Department of Pharmacology (Clinical Pharmacology), Showa University School of Medicine, 6-11-11 Kitakarasuyama, Setagaya-ku, Tokyo, 157-8577 Japan

**Keywords:** Methamphetamine, Methoxyphenamine, Smoke, Active inhalation, Passive inhalation, Urinary concentration

## Abstract

**Purpose:**

Methamphetamine (METH) is commonly abused through smoking. However, the lack of evidence regarding differences in urinary METH excretion after its active and passive inhalation has resulted in complications where the accused claims passive exposure. This study aimed to determine the differences in urinary excretion after active and passive inhalation of the drug, using methoxyphenamine (MPA) as a model for METH.

**Methods:**

Body temperature and locomotor activity were measured in mice as indicators of central nervous system toxicity. Six healthy adult male subjects were exposed to passive or active inhalation of MPA smoke in a small room, and urine samples were taken. MPA concentrations were measured using liquid chromatography–tandem mass spectrometry (LC–MS/MS).

**Results:**

There were no signs of toxicity in mice exposed to MPA smoke, ensuring the safety of the clinical study. Urinary MPA concentrations were significantly lower with passive inhalation compared with those of active inhalation. The maximum urinary MPA concentration in passive inhalation was 13.4 ng/mL, which was 1/60 of active inhalation with 800 ng/mL. The urinary excretion in passive inhalation until 24 h was 8.21 μg, which was 1/76 of active inhalation with 625 μg.

**Conclusions:**

Since METH and MPA are expected to be excreted similarly, urinary METH concentrations in passively exposed persons are expected to be lower than the cutoff value of the screening kit. If the urine screening test is positive, the suspect should be considered a METH user.

*Trial registration number*: jRCTs031210604, registration date: Feb. 9, 2022.

**Supplementary Information:**

The online version contains supplementary material available at 10.1007/s11419-022-00658-2.

## Introduction

According to a 2020 survey conducted by the United Nations Office on Drugs and Crime, amphetamine-type stimulants (ATS), along with marijuana and opioids [1], are widely abused worldwide. The estimated number of ATS users aged 15–64 was reported to be approximately 34 million, which represents 0.7% of the world population. ATS seizures have continued to increase, reaching a high record of 525 tons, a 15% increase compared with the previous year. In particular, seizures owing to methamphetamine (METH), the most psychoactive ATS, have increased approximately fivefold in the last 10 years, and METH abuse is still a serious social problem [2].

METH is taken up into presynaptic membranes via dopamine (DA) transporters and partially inhibits DA reuptake. It leads to an excessive release of newly generated DA into the synaptic cleft causing an increase in the DA concentration in the synaptic cleft and stimulation of DA receptors in the postsynaptic membrane [3]. Continued use of METH induces functional changes in dopaminergic reward pathways, leading to drug addiction [4]. In addition, METH increases serotonin and noradrenaline levels in the synaptic cleft and elicits symptoms caused by an excess of these monoamines [4]. Low to moderate doses (5–30 mg) of METH have been reported to cause euphoria, elevated mood, tachycardia, hypertension, and increased body temperature, whereas frequent and high doses induce psychosis [5].

METH can be abused in a variety of ways including intravenous injection, oral ingestion, snorting, and rectal administration [6]. While intravenous injection creates a more intense and immediate “high” than other methods, it also leaves injection marks on the arm which increases the risk of being arrested. As a result, consumption through inhaling the smoke produced by heating METH crystals became widespread in the mid-1990s [7], even among the younger generation [8]. Smoking METH has become the most common route of intake in recent years [9–11], because it leaves no injection marks and the user feels the effects immediately [12, 13]. In Japan, prosecutions for possession and/or use of METH continue to be the most common drug offenses, and the amount of METH seized in recent years has remained high [14], despite the emergence of a number of new psychoactive substances.

After absorption, a portion of METH is metabolized to amphetamine (AMP), and *p*-hydroxymethamphetamine via aromatic ring hydroxylation, and *N*-demethylation, mainly by CYP2D6 in the liver [15, 16]. Approximately 20–50% of METH is excreted via urine in its unchanged form, 15% as *p*-hydroxymethamphetamine, and 3% as AMP in the first 24 h [13, 16, 17]. Therefore, the detection of METH and its metabolite AMP in biological samples such as blood and urine is necessary to prove METH use for prosecution.

Clinical studies in which subjects actively inhaled METH smoke indicated that METH was detected in the blood and urine of the subjects [12, 13]. In some cases, METH was detected in the urine of persons who had not ingested it [18–20]. These cases suggest that passive exposure to METH smoke may result in the excretion of METH and possibly its metabolites in urine. However, the difference in urinary concentrations between active and passive inhalation of METH smoke is not clear. As a result, suspects occasionally claimed in court that the detection of METH in their urine was the result of passive exposure to METH, and they were eventually acquitted in some cases [21]. To prevent such confusion in court, clarification on the differences in urinary concentrations between active and passive inhalation of METH smoke is required. Clinical studies using METH are not legally and ethically allowed in Japan. Therefore, we investigated the possibility of using methoxyphenamine (MPA), which is a non-regulated and structural analog of METH, as a model drug for METH in mice [22]. We found that mice exposed to METH or MPA smoke exhibited similar patterns of urinary excretion of the unchanged form. Although there were differences in the amount of metabolites excreted between the two drugs, similarities were observed in the course of excretion over time. In addition, urinary drug concentrations after simulating passive exposure to METH or MPA smoke were significantly lower than those after the active exposure in mice. These results indicate that MPA could be a useful model drug for the study of METH inhalation.

While MPA is administered orally to humans as a cough suppressant [23], there are no reports of its administration by inhalation. In general, psychoactive drugs absorbed through the lungs are easily transported to the brain without first-pass effects and are, therefore, thought to act on the brain more quickly and profoundly than those administrated orally [24, 25]. However, there is little information on the toxicity of MPA inhalation, particularly its effect on the central nervous system (CNS).

The purpose of this study was to clarify whether the pattern of urinary drug excretion differs between active and passive METH exposure, using MPA as a model drug. At the beginning of this study, we examined the effects of MPA inhalation on the CNS in mice to ensure the safety of the subsequent clinical study. We then examined urinary drug concentrations in subjects exposed actively and passively to MPA smoke.

## Materials and methods

### Reagents

MPA hydrochloride and diazepam-d5 were purchased from Sigma-Aldrich (St. Louis, Missouri, USA). *O-*Desmethylmethoxyphenamine (ODMP) was purchased from Chem Space (Riga, Latvia). QUECHERS Q-sep Q150 Extraction Packet (#25851; magnesium sulfate 6 g, sodium acetate 1.5 g) was purchased from Restek Corporation (Bellefonte, PA, USA). Butorphanol tartrate was purchased from Meiji Seika Pharma (Tokyo, Japan). Medetomidine hydrochloride was purchased from Nippon Zenyaku Kogyo (Fukushima, Japan). Midazolam was purchased from Maruishi Seiyaku (Osaka, Japan). Other common reagents used in this study were of the highest grade commercially available.

### Animal experiments

#### Animal and experimental design

**Fig. 1:**
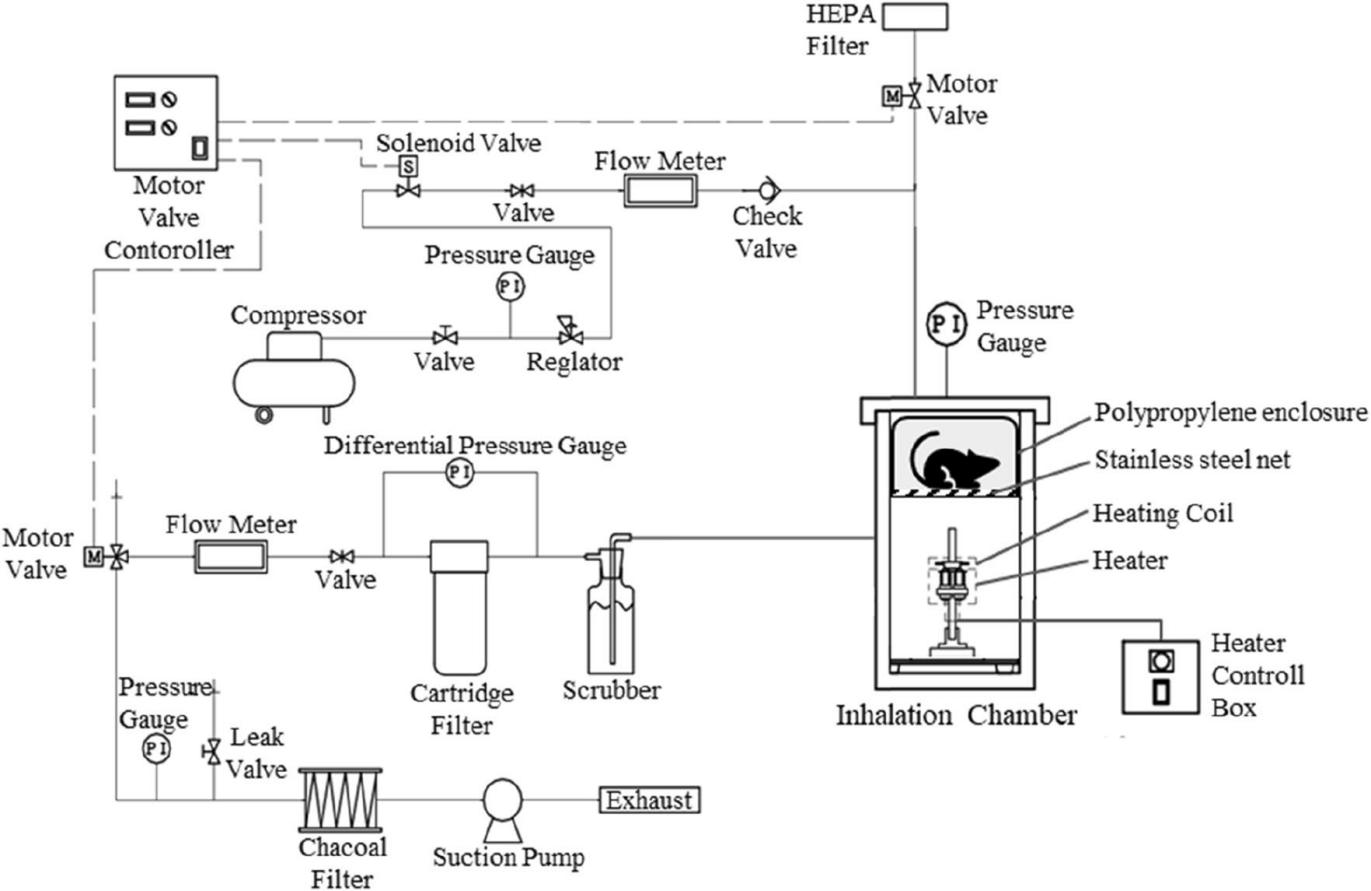
Inhalation chamber unit used for inhalation of MPA. Mice were exposed to MPA smoke in the inhalation chamber. The inhalation chamber had a volume of 2.5 L and a drug-heating coil at the bottom. A stainless steel net was placed in the middle of the chamber, and mice were placed on it during exposure

Male ICR mice aged 6 weeks were obtained from Sankyo Lab Service (Tokyo, Japan). Mice were housed in plastic cages placed in a temperature-controlled room (22 ± 1 °C) and maintained on a 12 h light–dark cycle with free access to food and water. The animals were exposed to MPA smoke using the multipurpose inhalation chamber unit [2.5 L (Fig. 1), Shibata Biotechnology, Tokyo, Japan]. Since 20–100 mg is supposed to be the dose of a one-time usage of METH being abused, the environment of active exposure was simulated by placing 50 mg of MPA (as hydrochloride salt) on a heating coil in the inhalation chamber and heating it to 600 °C to completely sublimate and generate MPA smoke. The mice were allowed to stay just above the heating area in the inhalation chamber to inhale the most concentrated MPA smoke (Fig. 1). MPA was heated for 1 min, left for 4 min, and then the air in the chamber was replaced with fresh air over a 2.5 min period. The air flow rate into the chamber was set at 18 L/min during ventilation. The air in the chamber was passed through a gas cleaning bottle filled with water and a charcoal filter twice before being discharged outside. The control mice were exposed to the smoke produced by heating 50 mg of lactose instead of MPA.

#### Measurement of body temperature and locomotor activity on the running wheel

Mice were anesthetized with a combination of medetomidine (0.375 mg/kg), midazolam (2 mg/kg), and butorphanol (2.5 mg/kg). Nano-Tag (Kissei Comtec Co., Matsumoto, Japan), a device measuring body temperature and activity, was implanted in the back of the mice at least 48 h before initiating the experiments. In measurement with the Nano-Tag device, activity was defined as cross-count data, providing a count of the number of times the XYZ acceleration vector synthesized waveform crosses the threshold levels from bottom to top per recording interval [26].

To compare with inhalation experiments, MPA at doses of 1 mg/kg (equivalent to the medication dose [27]) or 5 mg/kg (equivalent to the inhaled dose in mice calculated based on the chamber air concentration, breath times, and breath volume) were administered orally to the mice. Tap water (10 mL/kg) was administered orally to the control mice. Eighteen mice were divided into three groups: group A (inhalation), group B (1 mg/kg, *p.o.*), and group C (5 mg/kg, *p.o.*). On the day before the experiment, mice were placed on a running wheel (15 cm diameter, 5 cm track width) for 30 min to acclimate them to the tool [28–30]. On day one, group A was exposed to 50 mg of lactose smoke, and tap water was orally administered to group B and C as a control condition. On day two, group A was exposed to 50 mg of MPA smoke, and 1 mg/kg and 5 mg/kg of MPA was orally administered to group B and C, respectively. On both days, the mice were immediately placed on the running wheel for 45 min after inhalation exposure or oral administration. The body temperature and locomotor activity were analyzed by the Nano-Tag viewer program (Kissei Comtec Co.) [31].

### Clinical study

#### Study design

**Fig. 2:**
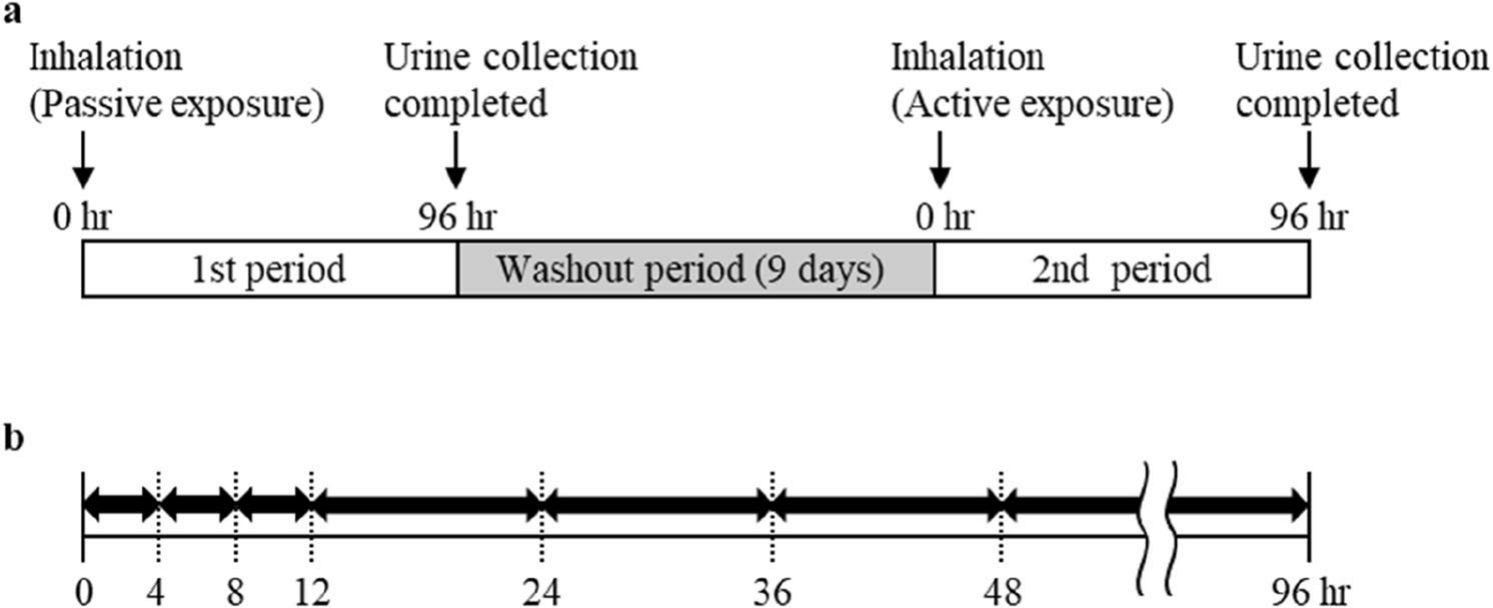
Schedule of the clinical study and urinary collection. Schedule of 1st and 2nd periods of clinical study (**a**) and urinary collection (**b**). Schedule of urinary collection was applied to the 1st and 2nd periods of this study. Arrows pointing both directions indicate the urinary collection period during 96 h

The clinical study was designed to assess whether subjects’ urine concentrations of MPA and its metabolites differed after active and passive inhalation of MPA simulating the smoking of METH in a car. This was a single-arm, open-label, and crossover study conducted between February 28 and March 18, 2022. The study consisted of two parts: the 1st period which involved passive inhalation and the 2nd period which involved active inhalation of MPA smoke (Fig. 2). To simulate the environment of passive and active inhalation in the car, both periods were conducted in a 4.3 m^3^ greenhouse, equivalent to the internal volume of a sedan car. Similar to the animal experiments, 50 mg of MPA was completely sublimated on a heating coil at 600 °C for 1 min.

The 1st period was conducted to simulate a situation in which the passively exposed person was in the passenger seat next to the smoke source (actively inhaling person) in the driver's seat. Subjects were seated at a distance of 80 cm horizontally from the MPA heating site and inhaled the smoke through their nose with resting breaths. The air inside the greenhouse was stirred by a fan to equalize the smoke concentration inside. Inhalation lasted 300 s after the start of heating and the subjects were moved to the fresh air environment. Urine was collected for 96 h after inhalation, and a washout period of 9 days was allowed before the 2nd period was performed.

In the 2nd period, the active inhalation was simulated: the open end of a paper cylinder (10 cm vertically) was placed just above the MPA heating element, and the other end was placed near the subjects’ nose to prevent smoke leakage. Subjects inhaled the smoke through their nose with effort for 90 s followed by resting breaths from 90 to 300 s of heating. Inhalation lasted 300 s after the start of heating and the subjects were moved to the fresh air environment. Urine was collected for 96 h after inhalation.

#### Study subjects

The subjects were Japanese adult males who were considered to be in good health by a physician based on vital signs (blood pressure, heart rate, and body temperature), electrocardiogram, medical interview, and results of medical examinations performed within the past year. The inclusion criteria were as follows: 20–49 years old, body mass index (BMI) 18–30 kg/m^2^, non-smoker, no drug or food allergies, and no intake of dietary supplements.

#### Sample collection, preparation, and extraction

In the 1st and 2nd study periods, urine was collected as a pre-sample before MPA smoke inhalation. Total urine samples were collected every 4 h until 12 h and every 12 h from 12 to 96 h after MPA smoke inhalation (Fig. 2). After the urine volume for each collection was measured, 10 mL of urine was stored in polypropylene tubes at − 20 °C until urinary drug concentrations were determined.

MPA was extracted from the urine samples by adding 200 mg of QuEChERS salt, 600 μL of acetonitrile, and 10 ng of diazepam-d5 as internal standard (IS) to 200 μL of urine and stirring vigorously for 10 min. After centrifugation at 15,000 × g for 10 min, the organic phase was collected. Then, after the evaporation of the solvent under a nitrogen stream, the residue was dissolved in 200 μL of methanol and subjected to liquid chromatography–tandem mass spectrometry (LC–MS/MS) analysis. Calibration curves were prepared by adding MPA or its metabolite ODMP to blank urine at concentrations of 0.1, 0.25, 0.5, 1, 5, 25, 50, and 100 ng/mL. If the drug concentration in the subject’s urine sample exceeded the range of the calibration curve, the urine sample was diluted with blank urine and the extraction and concentration measurements were performed again. Urinary drug excretion was determined by multiplying the urinary drug concentration by the urine volume.

### LC–MS/MS analysis

#### LC–MS/MS conditions

An LC–MS/MS system consisting of LC-40ADXR and LCMS-8045 (Shimadzu Corporation, Kyoto, Japan) was applied for the measurement. Chromatographic separation was achieved on a Phenomenex Kinetex XB-C18 column (2.1 mm ID × 100 mm, 2.6 μm; Shimadzu) with an equivalent Phenomenex Security Ultra C18 guard column (2.1 mm ID; Shimadzu). The column temperature was set at 40 °C. The mobile phases were (A) 10 mM ammonium formate and 0.1% formic acid, (B) methanol containing 10 mM ammonium formate and 0.1% formic acid. The gradient started at 5% B, increased to 95% over 7.5 min, was held for 2.5 min, then immediately returned to 5%, and then held for 5 min. The flow rate was set at 0.3 mL/min and injection volume was 2 μL. After ionization with electrospray ionization (ESI) in positive mode, the samples were analyzed in multiple reaction monitoring mode (MRM). Flow rate of the nebulizer gas, the drying gas, and the heating gas were set at 3 L/min, 10 L/min, and 10 L/min, respectively. Temperatures of the interface, the desolvation line, and the heat block were set to 300 °C, 250 °C, and 400 °C, respectively. The correlation coefficient and determination coefficient for the calibration curves of MPA and ODMP were in the range of 0.998 to 0.999, indicating that the calibration curves for MPA and ODMP showed good linearity. The instrumental conditions for each compound are indicated in Table S1.

#### Measurement of MPA concentration in the air of animal and clinical studies

In the animal study, MPA was heated under the described conditions, and a 10 mL portion of the air in the chamber was collected by a gastight syringe 1 min after heating began. The collected gas was absorbed in 2 mL methanol by shaking in a hermetically sealed vial for 10 min and analyzed using LC–MS/MS.

In the clinical study, MPA was heated under the indicated conditions. A 10 mL portion of the air was collected by a gastight syringe at the subject’s inhalation position at each of the 1st and 2nd periods in the greenhouse at 10, 20, 30, 60, 90, 120, 150, 180, 240, and 300 s after heating began. The collected gas was absorbed in 2 mL methanol by shaking in a hermetically sealed vial for 10 min and analyzed using LC–MS/MS. Calibration curves were prepared at nominal concentrations of 0.1, 0.25, 0.5, 1, 5, 25, 50, and 100 ng/mL for MPA.

### Statistical analysis

Differences in the data of body temperature and locomotor activity on the running wheel between MPA treated and control condition mice were analyzed using paired *t*-tests. Differences in the data of body temperature and locomotor activity on the running wheel after MPA administration between the inhalation and 5 mg/kg *p.o.* and between the 1 mg/kg *p.o.* and 5 mg/kg *p.o.* groups were analyzed using Student’s *t*-tests. Differences in the data of urinary drug concentration and urinary drug excretion after MPA inhalation in the clinical study were analyzed using the Wilcoxon signed-rank test. All statistical analyses were performed on JMP Pro 16.0.

## Results

### Animal experiments

**Fig. 3:**
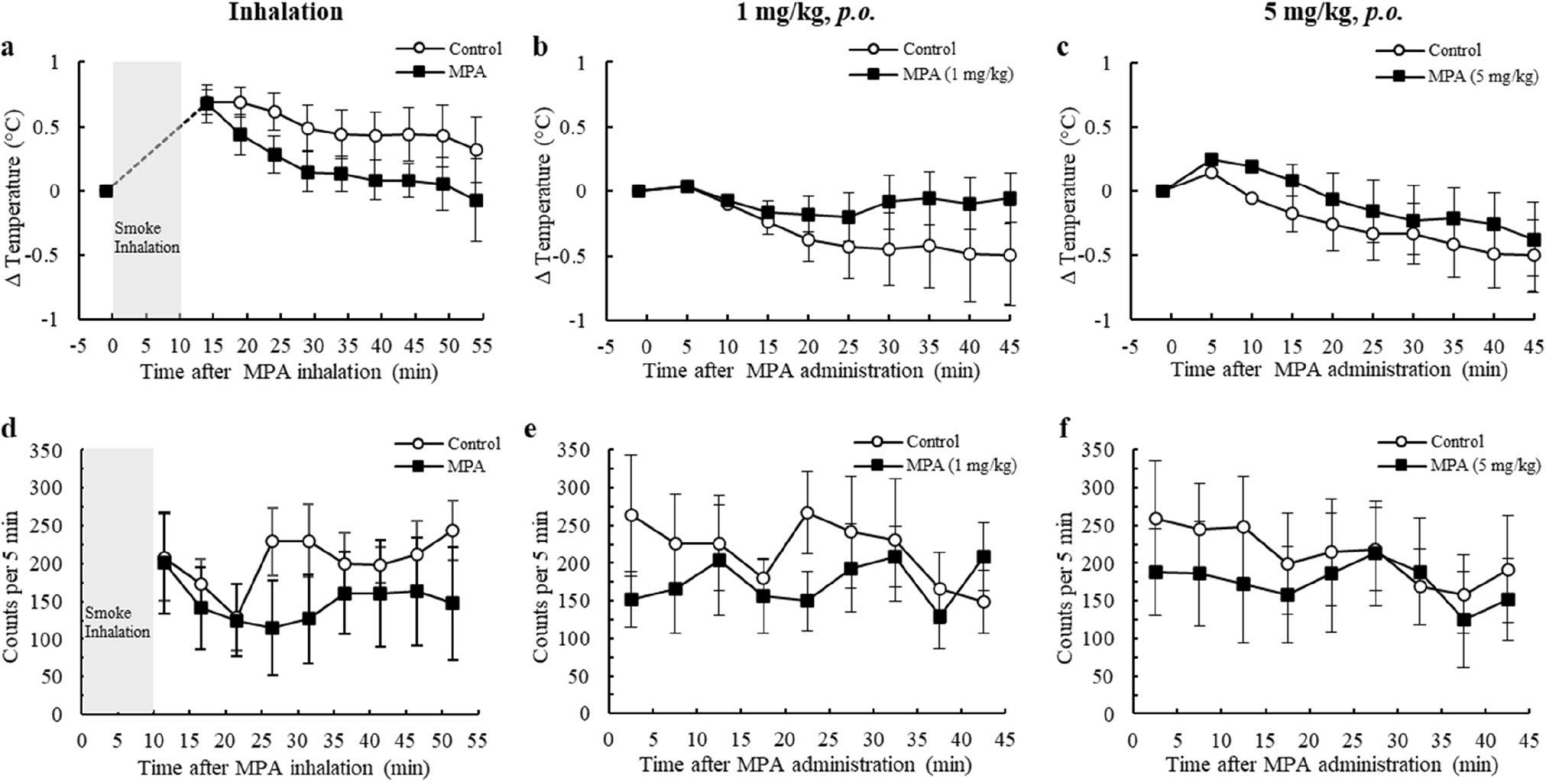
Effects of smoke inhalation or oral administration of MPA on body temperature (**a**–**c**) and locomotor activity on the running wheel (**d**–**f**) in mice. Body temperature was measured every 5 min for 45 min after MPA smoke inhalation (**a**), 1 mg/kg MPA *p.o.* (**b**), and 5 mg/kg MPA *p.o.* (**c**), using an implanted Nano-Tag device. Locomotor activity was measured for 45 min after MPA smoke inhalation (**d**), 1 mg/kg MPA *p.o.* (**e**), and 5 mg/kg MPA *p.o.* (**f**), using an implanted Nano-Tag device. The vertical axis represents the number of counts for 5 min recorded by the 3-axis sensor. The control animals were exposed to lactose smoke or administered tap water. Values represent the mean ± SEM (*n* = 6). Statistical analyses between MPA administration and control conditions were performed using paired *t-*tests. Statistical analyses between inhalation and 5 mg/kg *p.o.* groups, and between 5 mg/kg *p.o.* and 1 mg/kg *p.o.* groups were performed using Student’s *t-*test

To estimate the toxicity of inhaled MPA smoke, we examined its effects on the CNS in mice. Body temperature and locomotor activity on the running wheel were measured in mice exposed to MPA smoke under conditions that simulated active inhalation of the drug's smoke. MPA was administered orally to compare if its effects varied depending on the route of exposure. Body temperature and locomotor activity changes after inhalation of MPA smoke were not significantly different from control conditions at any of the times observed (Fig. 3a, d). The MPA concentration in the chamber air, when filled with smoke, was 326 ± 182 μg/L (*n* = 3) 1 min after heating began. Based on the fact that the mice used in this study weighed 30–36 g and the ventilation volume of ICR mice has been reported to be approximately 3 mL/min/g [32], the amount of MPA inhaled in 5 min was estimated to be approximately 5 mg/kg. We orally administered 1 mg/kg MPA, a dose equivalent to the human clinical dose, and 5 mg/kg MPA, a dose equivalent to the condition of active inhalation, to the mice. Body temperature changes (Fig. 3b, c) and locomotor activity (Fig. 3e, f) after oral administration of 1 and 5 mg/kg MPA were not significantly different from the control conditions. Body temperature changes (Fig. 3c) and locomotor activity (Fig. 3f) in the 5 mg/kg *p.o.* group were not significantly different from those in the 1 mg/kg *p.o.* group (Fig. 3b, e). In addition, there were no statistically significant differences in body temperature changes and locomotor activity following MPA inhalation (Fig. 3a, d) and in the 5 mg/kg *p.o.* group (Fig. 3c, f). Because of the animal’s proximity to the heated area in the smoke exposure chamber, a transient increase in body temperature was observed immediately after heating began (Fig. 3a). However, this temperature increase recovered to the level before the exposure 30 min later. These results indicate that inhalation of MPA smoke, which simulates active inhalation of the drug’s smoke, does not have a pronounced effect on the CNS, similar to oral administration in mice. Therefore, the following clinical study of MPA inhalation was conducted.

### Clinical study

#### Subject background

Six subjects were registered, and they all completed the study. All the obtained data were used for subsequent analysis. The subjects’ age and BMI ranged from 27 to 37 years and 19.9–29.6 kg/m^2^, respectively. No adverse events occurred during the study.

#### MPA concentration in the air

The present clinical study consisted of 2 periods; the first period simulated passive inhalation and the second period simulated active inhalation of METH. Under the 1st period conditions, the environmental MPA concentration reached a maximum of 0.27 μg/L at 150 s of heating with a minor change in concentration between 60 and 300 s (Fig. S1a). Under the 2nd period conditions, the MPA concentration at the subject’s inhalation position increased quickly after heating began, reaching a maximum value of 787 μg/L after 60 s, and then decreased over time. However, by 300 s since the start of heating, the concentration was 0.76 μg/L, exceeding the maximum concentration in the 1st period (Fig. S1b). The maximum concentration in the air during the 1st period was approximately 1/2900 of that in the 2nd period.

#### Urinary excretion of MPA and ODMP

**Fig. 4:**
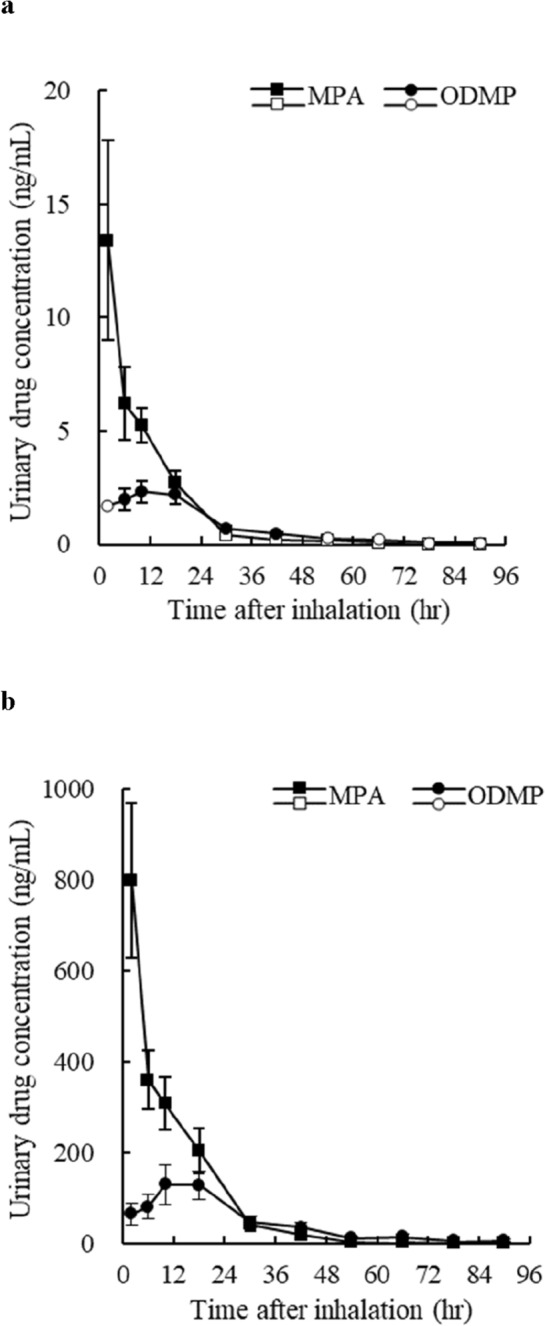
Urinary concentrations of MPA and ODMP. Changes in urinary drug concentrations after inhalation of MPA smoke in the 1st period (**a**) and 2nd periods (**b**). Values represent the mean ± SEM (*n* = 6). Intervals containing samples with urinary drug concentrations below the limit of quantitation are indicated by white marks. When the urinary concentrations of MPA and ODMP of the sample were above the limit of detection and below the limit of quantitation, 0.5 and 0.25 ng/mL were applied, respectively, and when below the limit of detection, 0 ng/mL was applied

In the 1st and 2nd periods of the study, subjects inhaled MPA smoke in the morning of day one, and urine samples were collected every 4 h until 12 h and every 12 h from 12 to 96 h after inhalation. Urinary MPA concentrations after inhalation of MPA in the 1st and 2nd periods were highest at 13.4 and 800 ng/mL, respectively, in the 0–4 h urine, and then decreased over time (Fig. 4, Table S2). Urinary MPA concentrations in the 1st period were below the lower limit of quantification (< 0.5 ng/mL) in some samples after 24 h, and in all samples after 60 h of inhalation (Fig. 4a, Table S2).

Meanwhile, urinary ODMP, a metabolite of MPA, concentrations in the 1st period were below the lower limit of quantification (< 0.25 ng/mL) in some samples at 0–4 h and after 48 h, and in all samples after 72 h of inhalation (Fig. 4a, Table S2). Urinary ODMP concentrations reached a maximum of 2.33 and 130 ng/mL at 8–12 h in the 1st and 2nd periods of the study, respectively, and then decreased over time (Fig. 4, Table S2). In the 2nd period, MPA and ODMP were quantifiable in all samples until 96 h (Fig. 4b). The maximum urinary concentrations of MPA and ODMP in the 1st period were approximately 1/60 and 1/56 of that in the 2nd period, respectively, and urinary drug concentrations in the 1st period were significantly lower than those in the 2nd period throughout the study (Table S2). Neither MPA nor ODMP was detected in the pre-sample for the 1st and 2nd periods.

The area under the curve (AUC) of urinary MPA and ODMP until 96 h after inhalation of MPA was calculated from the results in Fig. 4. Note that the data for the 1st period were estimated slightly higher than the original values because the lower limit of quantification was applied to samples with urinary concentrations of MPA and ODMP above the limit of detection and below the lower limit of quantification. The AUC of MPA was 163 ± 32.0 ng h mL^−1^ (estimated value) in the 1st period and 10,400 ± 1720 ng h mL^−1^ in the 2nd period. The AUC of ODMP was 80.8 ± 15.3 ng h mL^−1^ (estimated value) in the 1st period and 4630 ± 1030 ng h mL^−1^ in the 2nd period. The AUC of MPA and ODMP in the 1st period of the study was approximately 1/64 and 1/57 of that in the 2nd period, respectively, similar to the ratio of the maximum urinary concentrations. The ratio of the maximum urinary concentrations of MPA and ODMP and the time it took to reach the maximum urinary concentrations were comparable between the 1st and 2nd periods of the study, indicating that the urinary excretion patterns of MPA and ODMP in the 1st and 2nd periods were similar (Fig. 4).

**Fig. 5:**
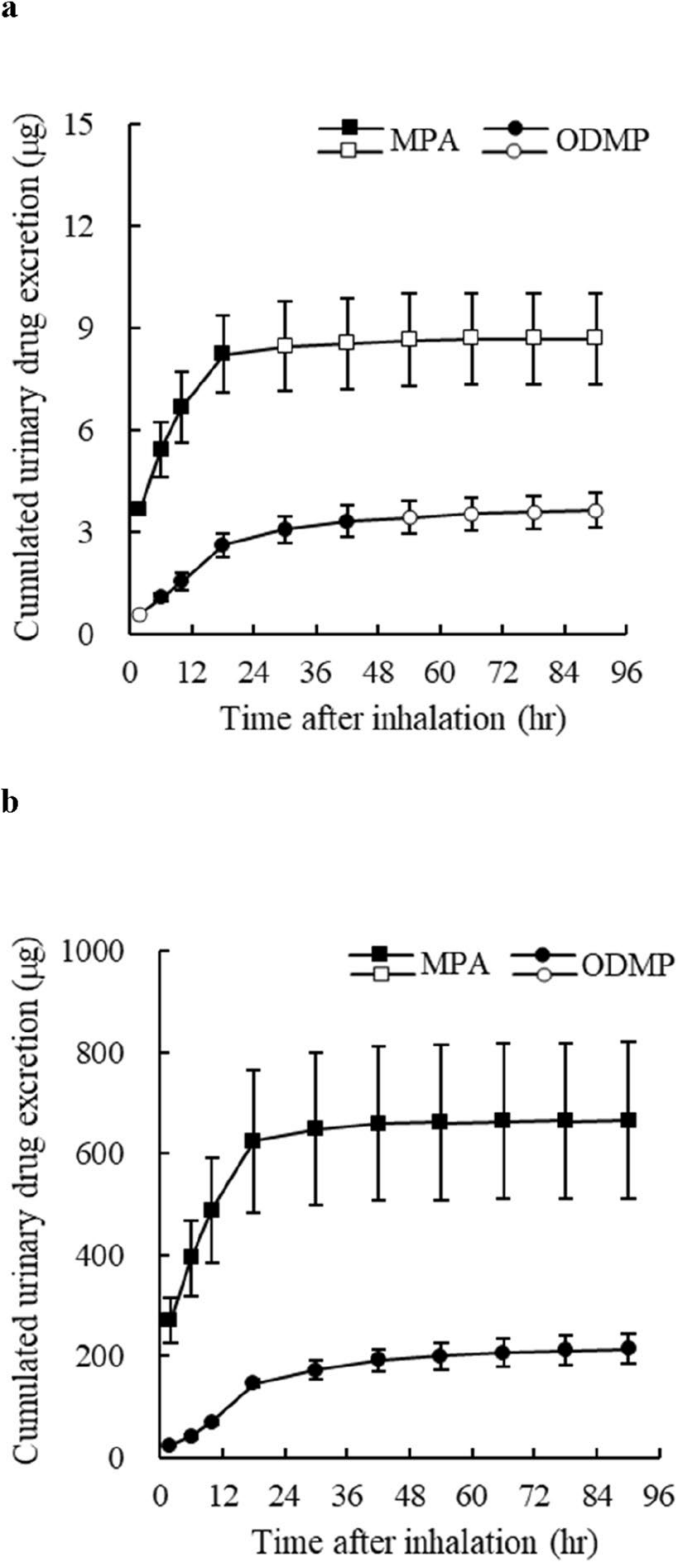
Cumulated urinary excretion of MPA and ODMP. Changes in total urinary drug excretion after inhalation of MPA smoke in the 1st period (**a**) and 2nd periods (**b**). Squares and circles indicate MPA and ODMP, respectively. Cumulated urinary drug excretion was calculated from the urinary drug concentration and volume in each interval. Values represent the mean ± SEM (*n* = 6). Intervals containing samples with urinary drug concentrations below the limit of quantitation are indicated by white marks. When the urinary concentrations of MPA and ODMP of the sample were above the limit of detection and below the limit of quantitation, 0.5 and 0.25 ng/mL were applied, respectively, and when below the limit of detection, 0 ng/mL was applied for calculations

Urinary excretion (Fig. 5, Table S3) for the 1st and 2nd periods of the study was calculated from urinary drug concentration (Table S2) and urinary volume (Table S4). By the end of 24 h, the MPA excreted was 8.21 μg in the 1st period and 625 μg in the 2nd period, and 8.68 μg (estimated value) in the 1st period and 666 μg in the 2nd period by 96 h (Fig. 5). By the end of 24 h, the ODMP excreted was 2.60 μg (estimated value) in the 1st period and 147 μg in the 2nd period, and 3.63 μg (estimated value) in the 1st period and 215 μg in the 2nd period by 96 h (Fig. 5). In the 1st period, urinary excretion of MPA and ODMP during the first 24 h was approximately 1/76 and 1/57 of that in the 2nd period, respectively, and urinary excretion in the 1st period was significantly lower than that in the 2nd period throughout the study (Table S3).

## Discussion

MPA is administered orally as a cough suppressant and has been used as a model drug for METH in clinical studies [33, 34], but there are no reports of its inhalation in humans. In general, smoke inhalation of drugs may facilitate its transfer to the brain and affect the CNS. Although no adverse effects on the CNS have been observed when therapeutic doses of MPA are administered orally, the effects of inhalation are not clear. If inhalation of MPA caused toxicity, effects on the CNS would be observed. In our previous study, a temporary and slight decrease in body temperature and locomotor activity were observed in mice exposed to MPA smoke, suggesting that its inhalation may have a mild depressive effect on the CNS [22]. Salbutamol, a β2-receptor agonist similar to MPA, has been reported to inhibit locomotor activity at high doses in rats and mice [35, 36]. While measuring locomotor activity in the open field is a good method for evaluating CNS stimulants such as METH [22, 37], it is not suitable for evaluating CNS depressants because mice acclimated to the environment are inactive and their body temperature tends to decrease [38]. Therefore, in this study, locomotor activity was measured using the running wheel, which is thought to be a superior method for evaluating depression of the CNS [30].

Body temperature changes and locomotor activity in mice when compared with the control conditions were not significantly different after inhalation of MPA with air concentrations simulating active inhalation or oral administration of the corresponding doses. In addition, body temperature changes and locomotor activity were similar between the 1 mg/kg *p.o.* group, which is equivalent to the human therapeutic dose, and the 5 mg/kg *p.o.* group, which is equivalent to inhalation under the present experimental conditions, and no CNS toxicity was observed with the increased dose. Body temperature changes and locomotor activity between the inhalation and 5 mg/kg *p.o.* groups were similar, and there were no differences in toxicity between inhalation and oral administration. Taken together, it is clear that MPA inhalation has little effect on the CNS, similar to oral administration, at least up to a dose equivalent to 5 mg/kg. Based on these findings, we considered MPA inhalation in humans to be feasible and conducted a clinical study.

Similar to METH, MPA is metabolized mainly by CYP2D6 [39, 40], undergoing *O*-demethylation and aromatic ring hydroxylation to produce ODMP and 5-hydroxymethoxyphenamine [41–43]. In our previous study, the total urinary excretion rate of METH and AMP was similar to that of MPA and ODMP after mice were exposed to smoke generated by heating METH or MPA, and the urinary excretion patterns were also similar [22]. Therefore, in this study, urinary concentrations of MPA and ODMP were measured after inhalation of MPA to estimate the changes in urinary concentrations of METH after its inhalation. The maximum urinary concentration ratio of MPA to ODMP was approximately 6:1 in the 1st and 2nd periods in this study (Fig. 4). The maximum urinary concentration ratio of METH to AMP is reported to be approximately 7:1 when 30 mg of METH (as hydrochloride salt) was heated in a glass pipe and the smoke generated was actively inhaled [12]. Therefore, it is indicated that the concentration ratio of MPA and ODMP excreted in the urine after inhalation is resemble to that of METH and AMP [12].

When MPA is administered orally to humans, the urinary excretion rate of the unchanged form up to 24 h is reported to be 18–31% [42, 43], which values are very close to the case of METH administered orally where 18–27% were excreted [16]. When METH is actively inhaled, the urinary excretion rate up to 72 h is reported to be approximately 37% [12], which is comparable to the urinary excretion rate when METH was administered orally [16]. The urinary excretion rate of MPA in mice during 24 h after active inhalation was about 20% [22], which is comparable to the urinary excretion rate when MPA was administered orally to humans [42, 43]. These reports suggest that the urinary excretion rates of the unchanged form after inhalation of MPA and METH are comparable. In this study, it was difficult to determine the amount of MPA inhaled by the subjects and it was not possible to calculate the exact urinary excretion rate. However, we observed similar drug concentrations in the air of the exposure chamber when 50 mg of METH or MPA was heated [22], suggesting that there is no difference in the vaporization of the two drugs. If METH was inhaled in the same way as MPA and the urine volume was similar, the urinary drug concentrations would be comparable. Considering these previous reports and the results obtained in this study, we conclude that MPA is a good model drug for studying urinary pharmacokinetics, including metabolites, in the human inhalation study. It was also indicated that it is possible to estimate the urinary METH concentrations of people who smoked the drug passively and actively from the results of this clinical study by substituting the urinary concentrations of MPA with METH.

The highest urinary METH concentrations reported was approximately 4500 ng/mL, and a concentration of approximately 1500 ng/mL was observed during 24–48 h after active inhalation of METH [12]. Since MPA concentrations of the air measured at the inhalation position of subjects in the 2nd period were as high as expected (Fig. S1), urinary MPA were estimated to be comparable to METH concentrations previously reported. However, urinary MPA concentrations were significantly lower than reported METH concentrations where METH smoke was inhaled. This is the first in human study where MPA was administered by inhalation, the smoke was inhaled through the nose rather than the mouth for safety reasons. The nose has a lower maximum inspiratory flow rate than the mouth [44], and therefore, the inhalation volume in the present study would be lower than that of the previous report in which METH was inhaled through the mouth. Although the subjects were asked to inhale as actively as possible, subjects complained that MPA smoke had a distinct and irritating smell, which might make it difficult to inhale through the nose actively. The active inhalation environment in this study was different from the actual abuse environment, and thus could result in lower urinary concentrations of MPA compared to METH.

Currently, the cutoff values or the detection limit for METH screening kits such as AccuSign and Signify ER often used for the screening assays are 500 or 1000 ng/mL. We revealed in this study that the maximum urinary concentrations of MPA and ODMP after passive inhalation of MPA were significantly lower than those after active inhalation in humans, at about 1/58 and 1/56 the amount, respectively (Fig. 4). Subjects with relatively low urine volume and high urinary MPA concentrations in the 1st period of this study, which simulated the passive inhalation condition, the maximum urinary MPA concentration at 0–4 h was 25.9 ng/mL, which was well below the cutoff value. There should be no substantial difference between passive exposure from the adjacent METH abuser and the passive inhalation conditions of MPA in this study. Even if there is some variation in urine volume among subjects, urinary concentrations are not expected to exceed the cutoff value. In the case of METH abusers, on the contrary, it is expected to detect METH well above the cutoff value for a longer time compared to the active inhalation of MPA shown in this study.

## Conclusions

This study revealed that urinary MPA concentrations after passive inhalation of MPA were well below the cutoff value and significantly lower than those after active inhalation. The urinary METH concentration after passive inhalation of METH is estimated to be similar to the urinary MPA concentration obtained in this study. Therefore, even in a confined space such as inside a sedan car, urine from a person who passively inhaled METH would show negative results on a screening kit. In other words, if a person tests positive on a screening kit, he or she could be considered an active METH user.

## Supplementary Information

Below is the link to the electronic supplementary material.

Supplementary file 1 (DOCX 84 KB)

Supplementary file 2 (XLSX 23 KB)

Supplementary file 3 (XLSX 37 KB)

## Data Availability

All data generated or analyzed during this study are included in this published article and its supplementary information files.
