# Supplementary material for: Urinary profiles of methoxyphenamine and its metabolite after inhalation of methoxyphenamine smoke in humans: aiming to distinguish between active and passive exposure
Source: Forensic Toxicol. 2023 Jan 6;41(2):230–40. doi: 10.1007/s11419-022-00658-2 (PMC10310607; doi:10.1007/s11419-022-00658-2)
Supplement: Supplementary file 1 — (DOCX 84 KB) [file 11419_2022_658_MOESM1_ESM.docx]

**Supplementary material**

**Urinary profiles of methoxyphenamine and its metabolite after inhalation of methoxyphenamine smoke in humans —Aiming to distinguish between active and passive exposure.**

**Forensic Toxicology**

Haruka Morinaka^1^, Asuka Kaizaki-Mitsumoto^1^, Hokuto Morohoshi^2,3^, Naoki Uchida^4^, Satoshi Numazawa^1^

1 Division of Toxicology, Department of Pharmacology, Toxicology and Therapeutics, Showa University School of Pharmacy, 1-5-8 Hatanodai, Shinagawa-ku, Tokyo, 142-8555, Japan

2 Clinical Research Institute for Clinical Pharmacology and Therapeutics, Showa University, 6-11-11, Kitakarasuyama, Setagaya-ku, Tokyo, 157-8577, Japan

3 Department of Hygiene, Public Health and Preventive Medicine, Showa University School of Medicine, 1-5-8 Hatanodai, Shinagawa-ku, Tokyo, 142-8555, Japan

4 Department of Pharmacology (Clinical Pharmacology), Showa University School of Medicine, 6-11-11 Kitakarasuyama, Setagaya-ku, Tokyo, 157-8577, Japan

**Corresponding author:**

Asuka Kaizaki-Mitsumoto

E-mail: asuka.0110@pharm.showa-u.ac.jp

Tel: 03-3784-8206　　Fax: 03-3784-8176

**b**

**a**


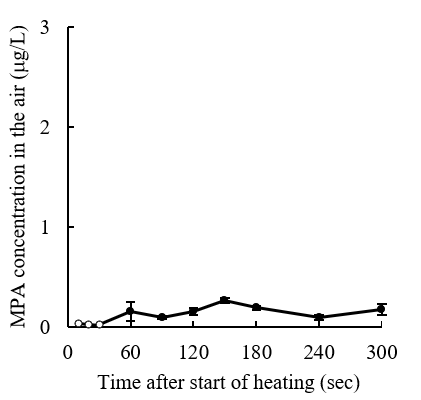

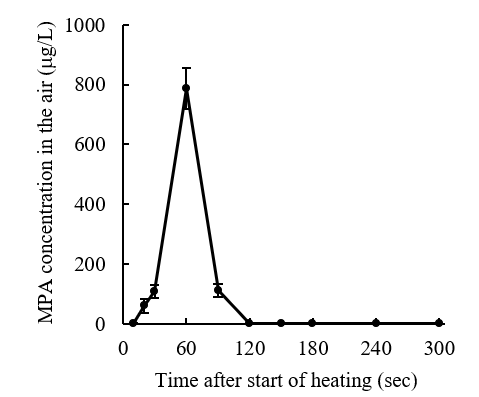
 **Fig. S1**  MPA concentrations in the air under clinical study conditions

Changes in the concentration of MPA in the air under the 1st period conditions (a) and the 2nd period conditions (b). Values represent the mean ± S.E.M. (n=3). Points containing samples below the limit of quantitation are indicated by white circles. When MPA concentrations of the sample were below the limit of detection, the value of 0 ng/mL was used to compute the mean values

**Table S1** Liquid chromatography-tandem mass spectrometry (LC-MS/MS) conditions

MRM: multiple reaction monitoring, CE: collision energy

**Table S2** Urinary concentrations of MPA and ODMP

Values represent the mean (range) (n=6). *a*, *b*, *c*, and *d* indicate the number of samples that were quantifiable, n=4, n=3, n=2, and n=1, respectively.

<LOQ: Below the limit of quantification, not calculated because urinary concentrations of all samples (n=6) were below the limit of quantification.

UDL: Below the limit of detection, not calculated because urinary concentrations of all samples (n=6) were below the limit of detection.

Statistical analysis was performed using the Wilcoxon signed test. When the urinary concentrations of MPA and ODMP of the sample were above the limit of detection and below the limit of quantification, 0.5 and 0.25 ng/mL were applied, respectively, and when below the limit of detection, 0 ng/mL was applied before statistical analysis. **p*<0.05 vs. 1st period

**Table S3** Urinary excretion of MPA and ODMP

Urinary drug excretion was calculated from the urinary drug concentration and urine volume in each interval. Values represent the mean (range) (n=6). *a*, *b*, *c*, and *d* indicate the number of samples that were quantifiable, n=4, n=3, n=2, and n=1, respectively.

NA: Not available, not calculated because urinary concentrations of all samples (n=6) were below the limit of quantification.

Statistical analysis was performed using the Wilcoxon signed test. When the urinary concentrations of MPA and ODMP of the sample were above the limit of detection and below the limit of quantification, 0.5 and 0.25 ng/mL were applied, respectively, and when below the limit of detection, 0 ng/mL was applied for calculations before statistical analysis. **p*<0.05 vs. 1st period

**Table S4** Urine volume of subjects

Values represent the mean (range) (n=6)
